# Overcoming the limitations of locally administered oncolytic virotherapy

**DOI:** 10.1186/s42490-019-0016-x

**Published:** 2019-07-01

**Authors:** JinWoo Hong, Chae-Ok Yun

**Affiliations:** 1 Department of Bioengineering, College of Engineering, Hanyang University, 222 Wangsimni-ro, Seongdong-gu, Seoul, 133-791 South Korea; 2 Institute of Nano Science and Technology (INST), Hanyang University, Seoul, Republic of Korea

**Keywords:** Oncolytic virus, Adenovirus, Nanomaterial, Local administration, Intratumoral injection, Polymer, Delivery systems

## Abstract

Adenovirus (Ad) has been most extensively evaluated gene transfer vector in clinical trials due to facile production in high viral titer, highly efficient transduction, and proven safety record. Similarly, an oncolytic Ad, which replicates selectively in cancer cells through genetic modifications, is actively being evaluated in various phases of clinical trials as a promising next generation therapeutic against cancer. Most of these trials with oncolytic Ads to date have employed intratumoral injection as the standard administration route. Although these locally administered oncolytic Ads have shown promising outcomes, the therapeutic efficacy is not yet optimal due to poor intratumoral virion retention, nonspecific shedding of virion to normal organs, variable infection efficacy due to heterogeneity of tumor cells, adverse antiviral immune response, and short biological activity of oncolytic viruses in situ. These inherent problems associated with locally administered Ad also holds true for other oncolytic viral vectors. Thus, this review will aim to discuss various nanomaterial-based delivery strategies to improve the intratumoral administration efficacy of oncolytic Ad as well as other types of oncolytic viruses.

## Background

Human serotype 5 adenovirus (Ad) is one of the most extensively evaluated gene transfer vector in various phases of clinical trials [1, 2]. Various genetic modification strategies can endow Ad with cancer specificity and make these vectors selectively lyse cancer cells through viral replication, ultimately generating an oncolytic Ad (oAd) [3]. oAd possesses several promising attributes that are beneficial for cancer gene therapy, such as high level of therapeutic transgene expression, facile production in high titer, ability to induce robust antitumor immune response, and transient nature of gene transfer with no risk of insertional mutagenesis [4, 5]. Notably, oAd, which is currently being marketed as Oncorine, was the first oncolytic virus to be approved for clinical use [6]. In support, numerous reports have demonstrated that locally administered oAds can induce potent antitumor effect in preclinical and clinical studies [7–13]. However, there are several notable limitations in clinical trials that restrict the therapeutic efficacy of oAds.

One of the limitations what we are facing in clinical setting is variable expression level of coxsackie Ad receptor (CAR), which is integral for internalization of human serotype 5 oAds [14, 15]. Heterogeneity of tumor population arising due to high mutational burden and rapid proliferation of cancer cells contributes to poor virion uptake at the time of administration and poor viral spreading through subsequent infection cycles [16–18]. Furthermore, some subset of tumors has been shown to progressively lose CAR expression with disease progression [19–21]. As most of clinical trials of new cancer drugs utilizes patients with advanced stages of illness, oAds must overcome this CAR-dependent internalization to elicit sufficient therapeutic efficacy. Indeed, 76.5% of oAds in ongoing clinical trials harbor diverse genetic modifications to the fiber region to enhance cellular uptake of the virus regardless of CAR expression level in heterogenic clinical tumor or tumor-specific internalization (Table 1). However, optimization and development of fiber-modified virus using genetic engineering is laborious, as improper modification can severely hinder viral replication, assembly, and overall production efficiency [8].

**Table 1:** Ongoing clinical trial of oncolytic adenovirus. The virus name and fiber modifications employed for enhancing internalization of various oncolytic adenovirus are presented

Trial ID	Phase	Disease Condition	Oncolytic adenovirus	Fiber modification	Internalization mechanism	Administration Route	Monotherapy/Combination therapy
NCT03029871	I	Lung cancer	Ad5-yCD/mutTKSR39rep-ADP	None	Coxsackie and adenovirus receptor (CAR)	Local delivery	Combination therapy
NCT02555397	I	Prostate cancer	Ad5-yCD/mutTKSR39rep-hIL12	None	CAR	Local delivery	Monotherapy
NCT03281382	I	Pancreatic cancer	Ad5-yCD/mutTKSR39rep-hIL12	None	CAR	Local delivery	Combination therapy
NCT03190824	II	Skin cancer	OBP-301	None	CAR	Local delivery	Monotherapy
NCT02045602	I	Multiple cancer types	VCN-01	replacement of the heparan sulfate glycosaminoglycan putative-binding site KKTK of the fiber shaft with an integrin-binding motif RGDK for tumor targeting	Integrin	Systemic delivery	Combination therapy
NCT03799744	I	Head and neck cancer	VCN-01	replacement of the heparan sulfate glycosaminoglycan putative-binding site KKTK of the fiber shaft with an integrin-binding motif RGDK for tumor targeting	Integrin	Systemic delivery	Combination therapy
NCT03225989	I/II	Multiple cancer types	LOAd703	Serotype 35 adenovirus fiber	CD46	Local delivery	Combination therapy
NCT02705196	I/II	Pancreatic cancer	LOAd703	Serotype 35 adenovirus fiber	CD46	Local delivery	Combination therapy
NCT03003676	I	Skin cancer	ONCOS-102	serotype 5/3 capsid-modified adenovirus	Desmoglein 2	Local delivery	Combination therapy
NCT03514836	I/II	Prostate cancer	ONCOS-102	serotype 5/3 capsid-modified adenovirus	Desmoglein 2	Local delivery	Combination therapy
NCT02879669	I/II	Pleural mesothelioma	ONCOS-102	serotype 5/3 capsid-modified adenovirus	Desmoglein 2	Local delivery	Combination therapy
NCT03852511	I	Multiple cancer types	NG-350A	group B Ad11p/Ad3 chimeric adenovirus	Desmoglein 2	Local & Systemic delivery	Monotherapy
NCT03714334	I	Brain cancer	DNX-2440	insertion of integrin binding RGD-4C motif for tumor targeting in fiber domain	Integrin	Local delivery	Monotherapy
NCT03178032	I	Brain cancer	DNX-2401	insertion of integrin binding RGD-4C motif for tumor targeting in fiber domain	Integrin	Local delivery	Monotherapy
NCT02798406	II	Brain cancer	DNX-2401	insertion of integrin binding RGD-4C motif for tumor targeting in fiber domain	Integrin	Local delivery	Combination therapy
NCT03896568	I	Brain cancer	BM-Ad5-DNX-2401(DNX-2401 loaded into mesenchymal stem cell)	insertion of integrin binding RGD-4C motif for tumor targeting in fiber domain	Integrin	Systemic delivery	Combination therapy
NCT03072134	I	Brain cancer	NSC-CRAd-Survivin-pk7(Neural stem cells loaded with CRAd-Survivin-pk7)	fiber protein polylysine modification (pk7) for enhanced cell internalization	Nonspecific enhancement in cell uptake through increased cationic charge (polylysine)	Local delivery	Combination therapy

Another limitation of oAd is its poor intratumoral retention and nonspecific shedding to surrounding normal tissues [14, 22]. A decent fraction of intratumorally administered oAd does not internalize into tumor cells and shedding of virions into the bloodstream eventually leads to nonspecific sequestration of viruses into normal tissues, thus leading to adverse side effects and insufficient therapeutic efficacy. Furthermore, these virions exposed to the blood stream can be easily recognized by host immune system to further accelerate the clearance of oAd through an induction of antiviral immune response [16]. Moreover, adverse inflammatory response can arise due to immune recognition of these oAd [23, 24]. Additionally, biological activity of intratumorally administered oAd in tumor tissues are relatively short, thus necessitating either multiple injections or large viral dose to achieve durable antitumor effect [25]. Thus, a novel delivery system that can enhance and prolong intratumoral retention of locally administered oAds is required.

A promising strategy to address these limitations of locally administered oAds is nanomaterial-based delivery system. For instance, masking of anionic Ad capsid via complexation with various cationic nanomaterials allowed Ad to be internalized into cells in a CAR-independent manner [26, 27]. Further, enhanced cellular internalization of oAd by nanomaterials can improve viral replication and elevate therapeutic transgene expression levels by oAd in cancer cells, thus greatly improving the therapeutic effect of armed oAds [26, 28]. Alternatively, gel- or magnetic nanoparticle (MNP)-based delivery of oAd have been shown to improve intratumoral retention of virus and limit the nonspecific shedding [14]. Although there are limited number of literature available, the nanomaterial-based local delivery platform has also been explored to address the limitations of other oncolytic viruses through similar principles as mentioned above [29]. Based on these premises, the present review aims to discuss how various nanomaterial delivery platforms for oAd (listed in Table 2) overcomes the conventional limitations of locally administered oAd in great detail and also explore how similar strategies are being explored for other oncolytic viral vectors.

**Table 2:** Nanomaterial-based delivery of oncolytic adenovirus. Nanomaterials with different attribute (cationic nanomaterials, hydrogels, and magnetic nanoparticles) can improve the local delivery efficiency of virus to tumor tissues. Table summarizes individual cases discussed in this review

No.	Nanomaterial	Characteristics of nanomaterial	Oncolytic adenovirus	Therapeutic transgene	Complexation Method	Reference
1	deoxycholic acid-conjugated PEI (DA3)	Cationic nanomaterial	RdB-KOX	Artificial transcriptional repressor zinc-finger protein specific to vascular endothelial growth factor (VEGF) promoter	Electrostatic interaction	[27]
2	mPEG-PEI-g-Arg-S-S-Arg-g-PEI-mPEG (PPSA)	Cationic/bioreducible nanomaterial	DWP418	Human relaxin	Electrostatic interaction	[24]
3	multidegradable bioreducible core-cross-linked PEI (rPEI)	Cationic/bioreducible nanomaterial	RdB/shMet	Short hairpin RNA specific to c-Met	Electrostatic interaction	[29]
4	PEGylated paclitaxel conjugated to arginine grafted bioreducible polymer (APP)	Cationic, bioreducible, and chemodrug-conjugated nanomaterial	oAd-vp53	p53 variant that is resistant to p53 inactivation	Electrostatic interaction	[32]
5	lithium 3-[2-(perfluoroalkyl)ethylthio]propionate) & 25 kDa branched PEI (PEI-Mag2)	Cationic/magnetic nanomaterial	dl520	No therapeutic transgene	Electrostatic interaction	[33]
6	polybrene-modified nanoparticles (PB-Mag1) & silica-modified nanoparticle decorated with branched PEI (SO-Mag2)	Cationic/magnetic nanomaterial	dl520	No therapeutic transgene	Electrostatic interaction	[34]
7	PEGylated and cross-linked iron oxide nanoparticles (PCION)	Cationic/magnetic nanomaterial	HmT	No therapeutic transgene	Electrostatic interaction	[35]
8	Injectable alginate gel	Biodegradable gel	DWP418	Human relaxin	Gel encapsulation	[36]
9	silk-elastin like protein polymer hydrogel (SELP hydrogel)	Biodegradable gel	Ad-C-Met	Short hairpin RNA specific to c-Met	Gel encapsulation	[37]
10	in situ cross-linkable gelatin hydrogels using gelatin-hydroxyphenyl propionic acid (GHPA gel)	Biodegradable gel	oAd-TRAIL	Secretable trimeric tumor necrosis factor-related apoptosis-inducing ligand (TRAIL)	Gel encapsulation	[38]
11	in situ cross-linkable gelatin hydrogels using gelatin-hydroxyphenyl propionic acid (GHPA gel)	Biodegradable gel	RdB/IL-12/GMCSF	Interleukin (IL)-12 and granulocyte-macrophage colony-stimulating factor (GM-CSF)	Gel encapsulation	[44]
12	gold nanorod (GNR)	Laser-induced hyperthermia	RdB-K35/KOX	Artificial transcriptional repressor zinc-finger protein specific to VEGF promoter	No complexation	[50]

## Main text

### Overcoming of CAR-dependent oAd uptake through cationic nanomaterials

An earliest report of oAd complexed with cationic nanomaterial to overcome oAd’s CAR-dependence by local administration approach was reported in 2014 [28]. In this report, a poly(ethyleneimine) (PEI) was conjugated with bile acid, generating DA3 polymer to improve cellular internalization of oAd into tumors with low CAR expression level. The group confirmed that increasing concentration of DA3 in respect to fixed concentration of Ad led to polymer concentration-dependent increase in the size and zeta potential of DA3-coated Ad (Ad/DA3), demonstrating that nanohybrid complex can be formed through electrostatic interaction between anionic viral capsid and cationic charge of DA3. Importantly, DA3 concentration-dependent improvement in Ad/DA3 transduction efficacy was observed in cancer cells regardless of cellular CAR expression level, thus allowing Ad/DA3 to be efficiently internalize into CAR-negative cancer cells. This high transduction efficacy of Ad/DA3 was achieved by increasing the quantity of virons internalized through endocytic vesicles and improving viral accumulation near the nuclear envelope (as well as the nucleus), suggesting that cationic nanomaterial can greatly improve cellular uptake and nuclear transport of virions. Importantly, oAd expressing anti-angiogenic gene (RdB-KOX) complexed with DA3 (RdB-KOX/DA3) elicited more potent anticancer effect in vitro and in vivo than naked RdB-KOX, leading to significant tumor growth inhibition up to 40 days post initial treatment due to more robust viral replication, induction of apoptosis, and anti-angiogenic effect in tumor tissues.

Another cationic nanomaterial, mPEG-PEI-g-Arg-S-S-Arg-g-PEI-mPEG (PPSA), have demonstrated similar improvement in intratumoral delivery of oAd by achieving CAR-independent internalization [26]. PPSA polymer is composed of low molecular weight PEIs and PEGylated to attenuate the polymer cytotoxicity while arginine (Arg) residues were incorporated to improve cellular penetration of the polymer. Ad complexed with PPSA (Ad/PPSA) showed polymer concentration-dependent increase in complex size and cationic charge, leading to improved transduction into both CAR-positive and -negative cancer cells. The improvement in transduction also translated into higher transgene expression level by the Ad/PPSA complex. Unlike Ad/DA3 complex from earliest report, the Ad/PPSA complex was bioreducible due to disulfide bond in polymer backbone, thus resulting in a low level of polymer cytotoxicity compared to 25 kDa branched PEI, a gold standard of transfection. Similar to Ad/DA3 complex, Ad/PPSA complex was internalized independently of CAR as pretreatment of cancer cells with Ad 5 fiber knob protein did not affect transduction efficacy of the complex as significantly as those of naked Ad. Notably, PPSA improved the anticancer effect of relaxin-expressing oAd (DWP418) to a greater extent than those of another arginine-grafted bioreducible polymer (ABP; previously shown to improve Ad transduction [30]) both in vitro and in vivo, showing that the careful designing of cationic nanomaterials with adequate chemical compositions are critical factors to improve therapeutic effect of oAds. Importantly, DWP418 complexed with PPSA (DWP418/PPSA) showed greater viral accumulation and induction of apoptosis in tumor tissues than naked DWP418, demonstrating that bioreducible cationic nanomaterials can improve the therapeutic efficacy of locally administered oAd while attenuating the polymer toxicity.

A multidegradable bioreducible core-cross-linked PEI (rPEI) with dendritic features also improved Ad transduction and antitumor efficacy of oAd [31]. rPEI incorporated multiple disulfide bonds among multiple low molecular weight PEIs to improve bioreducible aspect of the polymer in a similar manner as those of PPSA while endowing dendritic feature that has been reported to improve solubility and stability of polymers [32]. Both 16 kDa and 32 kDa rPEI, which possess different quantity of total PEI branches, exhibited markedly lower level of cytotoxicity and more efficiently improved the cellular internalization of Ad into cancer cells than 25 kDa branched PEI. c-Met inhibiting oAd complexed with 16 kDa or 32 kDa rPEIs elicited more potent cancer cell killing effect and higher level of viral replication than oAd complexed with 25 kDa branched PEI in cancer cells regardless of the cellular CAR expression level. These attributes of rPEI complexed oAds led to more robust suppression of c-Met as well, showing that multidegradable PEI can overcome CAR dependence, improve therapeutic transgene effect, and lower polymer-associated cytotoxicity.

Combinations of oAd with different conventional chemotherapeutics have been shown to improve the therapeutic efficacy of oAd [33, 34]. Paclitaxel (PTX) is a particularly promising candidate for oAd combination therapy, since PTX can improve multiple aspects of oAd, such as viral replication, transgene expression, and intratumoral virion distribution of oAd therapy. Most of these combination therapy studies have simply explored co-administration of PTX and oAd as separate therapeutic agents. In 2017, there was the first and only report exploring the synergistic antitumor efficacy of oAd complexed with PTX-conjugated cationic polymer, which concomitantly improved the therapeutic efficacy of both PTX and oAd as a single vector system [34]. In this report, PEGylated PTX was conjugated to ABP polymer to generate APP polymer, which was then complexed with oAd expressing a proapoptotic gene (oAd-vp53), to generate oAd-vp53/APP complex. A single complex was shown to simultaneously deliver both PTX and oAd to tumor tissues in an efficient manner. Similar to other cationic nanomaterials, complexation of Ad with APP greatly improved transduction into both CAR-negative and -positive cancer cells. Notably, oAd-vp53/APP complex elicited more potent cancer cell killing effect than naked oAd-vp53, oAd-vp53 complexed with ABP, APP treatment, or naked oAd-vp53 in combination with PTX (oAd-vp53 + PTX), showing that concurrent delivery of oAd and APP as a single hybrid vector can enhance the synergistic anticancer effect to a greater extent than a simple co-administration approach. Furthermore, the synergistic enhancement in cancer cell killing effect by oAd-vp53/APP complex was cancer-specific, since oAd-vp53/APP complex induced minimal level of cytotoxicity that was similar to PTX, oAd-vp53 + PTX, or APP treatment in several normal cell lines. Similar trends were observed in vivo where oAd-vp53/APP induced more potent tumor growth inhibition than naked oAd-vp53, PTX, APP, or oAd-vp53 + PTX treatments through an increase in viral replication, PTX accumulation, and induction of apoptosis in tumor tissues, showing that synergistic efficacy of combination therapy regimen can greatly augmented by a concomitant delivery via a single polymeric system.

### Enhancing tumor-specific accumulation of oAd through MNP-based delivery system

A magnet-guided internalization of oAd complexed with MNP was reported in 2010 [35]. In this report, a core-shell nanoparticle with magnetite core of about 10 nm was stabilized by a shell containing 68 mass % lithium 3-[2-(perfluoroalkyl)ethylthio]propionate) and 32 mass % 25 kDa branched PEI (PEI-Mag2), which was reacted with oAd, which is an E1A mutant Ad known as dl520, to generate a cationic and magnetic oAd-PEI-Mag2 complex through electrostatic interaction. oAd-PEI-Mag2 complex elicited more potent cancer cell killing effect than naked oAd, even when the complex was not guided by magnetic field (MF), in CAR-negative and multidrug-resistant cancer cells; likely due to net cationic charge of complex facilitating initial cellular uptake of oAd as reviewed in **Section 1**. The potent cell killing effect of oAd-PEI-Mag2 complex was greatly improved by guiding the complex with MF: this was due to (1) greater viral internalization by oAd-PEI-Mag2 (w/MF) than naked oAd (regardless of cellular CAR expression level) and (2) both PEI-Mag2 coating on oAd and MF improving the viral production ability of oAd-PEI-Mag2 complex. In contrast to promising in vitro findings, the tumor growth inhibition of naked oAd and oAd-PEI-Mag2 were statistically similar in most of the days examined by either the physical tumor volume measurement or luciferase imaging of luciferase-expressing tumors. The statistical difference between 2 groups was only observed on day 25 post initial treatment with luciferase imaging (no statistical difference was observed at this time point when tumor volume was obtained physically).

A subsequent report by the same group comparing oAd complexed with 3 different types of MNPs, including the original MNP (PEI-Mag2), showed more promising in vitro results [36]. Other than PEI-Mag2 from their initial report, two different MNPs [polybrene-modified nanoparticles (PB-Mag1) and silica-modified particles decorated with 25 kDa branched PEI (SO-Mag2)] were also utilized for comparison. oAd complexed with PB-Mag1 or SO-Mag2 (oAd-PB-Mag1 and oAd-SO-Mag2, respectively) improved internalization of virions into CAR-negative cancer cells in a similar manner as those observed by oAd-PEI-Mag2, and all 3 MNP-based nanocomplexes showed resistance to high concentration of serum content for cellular uptake. Although the enhancement in cellular uptake was the highest for oAd-PEI-Mag2 complex, both enhancement in oncolytic potency (IC_50_ of applied oAd/IC_50_ of applied MNP complex) and oncolytic productivity (IC_50_ of internalized oAd/IC_50_ of internalized MNP complex) was the highest for oAd-SO-Mag2, showing that initial enhancement in cellular uptake of oAd by MNP under MF does not necessarily translate to more potent oAd activity. Furthermore, oAd-SO-Mag2 also showed highest resistance toward anti-Ad neutralizing antibodies among all 3 MNP complexes: the oncolytic potency of oAd-PEI-Mag2 and oAd-PB-Mag1 complexes diminished by more than 8-fold in the presence of neutralizing antibodies, whereas only 2-fold reduction was observed for oAd-SO-Mag2 complex. In contrast to their initial report, both oAd-PEI-Mag2 and oAd-SO-Mag2 treatment resulted in greater tumor growth inhibition than naked oAd treatment at day 25 of initial treatment (only through luciferase imaging like their initial report). However, the difference of tumor growth inhibition efficacy between oAd-SO-Mag2 and oAd-PEI-Mag2 was not significant although in vitro results strongly suggested that oAd-SO-Mag2 would induce more potent antitumor effect in vivo. Although the in vitro results were promising with SO-Mag2 improving both oncolytic potency of oAd and providing better protection toward pre-existing Ad neutralizing antibodies than PEI-Mag2, the enhancement in antitumor efficacy was still insufficient in this report.

A first report of significant in vivo antitumor activity by oAd complexed with MNP was reported in 2015 where MNP composed of PEGylated and cross-linked iron oxide nanoparticles (PCION) was utilized [37]. Similar to abovementioned MNP-based delivery of oAd, the transduction efficacy of the GFP-expressing Ad complexed with PCION (Ad/PCION) was greatly improved even when the cells were briefly exposed to the complex with MF (15 min) in both CAR-negative and -positive cancer cells, showing up to 141-fold improvement in GFP expression and was superior to those achieved by naked dAd with longer treatment period (24 h exposure). Furthermore, PCION coating of hypoxia-responsive oAd (HmT) improved the cancer cell killing effect and viral production in both CAR-positive and -negative cancer cells under MF exposure; up to 2910-fold higher viral production for HmT-PCION than naked HmT. A single intratumoral injection with HmT/PCION without MF induced more potent tumor growth inhibition than naked HmT over the entire observation period (30 days post initial treatment), showing that HmT/PCION can internalize into tumor effectively due to net cationic charge of the polymer. This potent tumor growth inhibiting effect of PCION was further enhanced when used in combination with MF, showing that exposure of tumor to MF can facilitate internalization of oAd into tumor and enhance its accumulation. This was due to HmT/PCION being internalized more effectively into tumor than naked HmT due to cationic charge of the complex. Moreover, the therapeutic effect of the HmT-PCION complex was further improved by exposure to MF, leading to most robust tumor growth inhibition. Importantly, HmT/PCION without MF showed greater accumulation in the liver than tumor tissues likely due to nonspecific shedding of excess virion as described in **Introduction**, whereas intratumoral MF exposure for HmT/PCION treatment led to 450-fold enhancement in tumor-to-liver ratio compared to HmT/PCION without MF, showing that MNP-based oAd carrier with high sensitivity toward MF can greatly improve intratumoral retention and accumulation as well as suppressing nonspecific hepatic sequestration the virus to enhance both therapeutic and safety profile of oncolytic virotherapy.

### Enhancing tumor-specific accumulation and overcoming nonspecific shedding of oAd through gel-based delivery system

Locally administered oAd normally requires multiple injections to achieve durable and sufficient therapeutic efficacy due to nonspecific shedding of the virion to normal tissues, short biological activity of oAd in tumor tissues, and antiviral immune response leading to rapid clearance of virions. To this end, gel-based delivery of oAd is a promising strategy to overcome these limitations and prolong oncolytic activity of oAd in tumor tissues. A first report of such system was reported in 2013 where injectable alginate gel was used to deliver relaxin-expressing oAd (DWP418) into tumor tissues [38]. The biological activity of GFP-expressing Ad loaded in alginate gel was prolonged compared with naked Ad, leading to higher level of GFP being retained over an extended period as Ad was continuously released from the gel and retained in gel up to 11 days in vitro*.* Similarly, DW418 released from gel preserved their oncolytic activity to a greater extent than naked DWP418 being incubated under physiological temperature of 37 °C up to 9 days in vitro [oncolytic activity of naked DWP418 (17.6%) versus DWP418 from gel (80.6%)], showing that protective gel matrix can function as a depot system to release and protect oAd against physiological temperature. A single intratumoral injection of oAd-loaded gel (DWP418/gel) led to durable tumor growth inhibition in two different xenograft tumor models and elicited more potent antitumor effect than naked DWP418 up to 41 days post treatment (terminal point of observation): the tumor growth inhibitory effect was 38.2 and 74.3% for naked DWP418 and DWP418/gel, respectively. The initial antitumor activity by both naked DWP418 and DWP418/gel were similar in two xenograft tumor models during early periods (8 or 14 days post treatment), but the difference in tumor volume between two groups became greater with time, showing that gel-based delivery elicits anticancer effect in sustained manner. The durable antitumor response mediated by DWP418/gel was due to the virus being retained more efficiently in tumor tissues (up to 42-fold higher virion accumulation) at 15 to 24 days post treatment compared to naked DWP418. Immunohistochemical analysis of tumor tissues revealed that DWP418/gel treatment led to greater quantity of oAd being detected in both peripheral and central tumor regions, whereas the naked DWP418 treatment only led to virus being detected along the needle tracks in localized manner, showing that gel-mediated delivery of oAd both prolongs oAd accumulation and enhances viral distribution in solid tumors. Importantly, DWP418/gel significantly attenuated accumulation of virion in normal tissues, such as heart, kidney, stomach, liver, spleen, and the blood stream while it improved the intratumoral accumulation of DWP418, suggesting that sustained release of oAd from gel can attenuate nonspecific shedding of virion to normal tissues while enhancing its intratumoral accumulation (due to improved viral persistence and efficient replication of viron) to improve both the therapeutic and safety profile of locally administered oAd.

Similar results were achieved with a silk-elastin like protein polymer (SELP) hydrogel for the delivery of oAd in a different report [39]. This report also demonstrated that oncolytic activity of oAd expressing short hairpin RNA (shRNA) against c-Met released from SELP gel was retained at a higher level than naked oAd when both groups were incubated in PBS under physiological temperature up to 28 days in vitro (1.5- to 3-fold higher oncolytic activity from day 7–21 of incubation) while eliciting more potent and durable antitumor effect than naked oAd in vivo up to 28 days post treatment. SELP gel also enhanced virion retention and shRNA-mediated suppression of vascular endothelial growth factor (VEGF) expression in tumor tissues up to 3 weeks post treatment and attenuated Ad-associated hepatotoxicity, suggesting that both alginate and SELP gels can prolong intratumoral retention of virion while attenuating nonspecific shedding to the liver.

A first report of gel-based oAd delivery system in immunocompetent Syrian hamster model was reported in 2017 [40]. Syrian hamster model is replication-permissive to human serotype 5 Ad and provides a better insight into side effects and therapeutic efficacy of oAd than an immunocompetent murine tumor model [41–43]. Jung et al., reported of a system where a tumor necrosis factor-related apoptosis-inducing ligand (TRAIL)-expressing oAd (oAd-TRAIL) was encapsulated in gelatin-based hydrogel (oAd-TRAIL/gel) in this report to enhance and prolong the antitumor efficacy of oAd [40]. Hydrogel enhanced and prolonged intratumoral retention of oAd-TRAIL post infection while improving TRAIL-mediated induction of apoptosis and inhibition of tumor cell proliferation in the treated tumor. This report was also a first report to illustrate that gel stiffness plays an integral part in the regulation of viral release profile both in vitro and in vivo, showing that in vitro optimization of gel stiffness for release of oAd does not necessarily translate to prolonged antitumor effect in vivo; 3380 Pa stiffness was required for virus to be released in sustained manner in vitro compared to 50 Pa hydrogel, whereas 50 Pa hydrogel was optimal for the gel system to achieve sustained antitumor effect in vivo. These findings show that careful modulation of gel stiffness is necessary to allow efficient and prolonged release of oAd in vivo, as the viral quantity remaining in tumor tissues should be optimized to consider tumor growth rate. Similar to alginate and SELP gel, gelatin-based hydrogel also attenuated nonspecific shedding of virion to liver tissues (15.7-fold lower than naked oAd-TRAIL) while improving intratumoral quantity of oAd-TRAIL by 17.2-fold. Notably, oAd-TRAIL/gel greatly attenuated antiviral immune response to a basal level while preserving the ability of oAd to induce robust antitumor immune response; as evidenced by greater quantity of interferon (IFN)-γ secretion following co-culture of irradiated cancer cells with immune cells and intratumoral infiltration of CD4^+^ and CD8^+^ T cells following oAd-TRAIL/gel treatment than naked oAd-TRAIL. Their findings provided strong evidences that gel-based delivery system can attenuate oAd-associated antiviral immune response, which is detrimental for oncolytic virotherapy, by having the gel matrix functioning as a protective reservoir to shield the virus from host immune system while preserving the antitumor immunity of the virus.

A hydrogel-based system can also be utilized to release different therapeutic cargos, such as chemotherapeutics, proteins, and antibodies [44, 45]. To this end, a gelatin-hydroproxyphenyl propionic acid (GHPA)-based hydrogel has been utilized to potentiate the antitumor immune response mediated by concomitant delivery of immune stimulatory oAd, which co-expresses interleukin (IL)-12 and granulocyte macrophage colony stimulating factor (GMCSF), and therapeutic dendritic cells (DCs) [46]. This hydrogel-based delivery system concomitantly delivered both oAd and DCs in an efficient manner to tumor tissues over extended period. Their findings illustrate that both oAd and DC can be released in a sustained manner from the gelatin gel upon induction of gel degradation, and the biological activity of oAd released from the gel was preserved at a higher level than naked oAd over 6-day period under physiological temperature. Similarly, the viability of DCs being released from GHPA gels were much higher than those of naked DCs under physiological temperature from 3 to 6 days post incubation. Concurrent delivery of both oAd and DC by GHPA gel (oAd + DC/gel) showed more potent tumor growth inhibition and higher survival benefit than naked oAd, DC monotherapy, or combination of oAd and DC without gel (oAd + DC) in a syngeneic murine tumor model during the entire observation period; due to both oAd and DC being retained more effectively at a greater quantity within tumor tissues by oAd + DC/gel group. Sustained release of oAd + DC/gel also led to highest population of both activated exogenous and endogenous DCs being found in tumor tissues. This was likely achieved by (1) prolonged retention of exogenous DCs and immune stimulatory oAd in tumor tissues and (2) highest intratumoral expression of various antitumor cytokines like IL-12, GM-CSF, and IFN-γ by oAd + DC/gel group, improving the activation and recruitment of endogenous DC. Additionally, it should be noted that both oAd and DC were more extensively distributed in both peripheral and distal tumor sites in oAd + DC/gel group than oAd + DC group (the distribution was highly localized to a small region), showing that both DC and oAd can distribute more evenly through tumor by gel-mediated release. oAd + DC/gel treatment also led to most effective prevention of thymic atrophy, which is a contributor to immunosuppression in advanced stages of clinical tumors following extensive chemotherapy [47], showing that prolonged retainment of both immunotherapeutics in tumor tissues by a single gel system can potentiate antitumor immune response and attenuate tumor-induced immunosuppression.

Although there is no available report to date, a nanomaterial-based combination immunotherapy using oAd and immune checkpoint inhibitor (ICI) could be a promising extension of the approach discussed above and should be examined in the future. There is strong line of both pre-clinical and clinical evidences to suggest that administration of oncolytic virus into tumors can prime immunologically unresponsive ‘cold’ tumor microenvironment, which restricts the therapeutic efficacy of ICI monotherapy, to a ‘hot’ environment that promotes the influx of immune cells and cytokines, ultimately potentiating antitumor immune response by ICIs and other immunotherapeutics [48]. In support, several different oAds in combination with ICIs are currently being investigated in clinical trials (Table 1; NCT03799744, NCT03003676, and NCT02798406). As nanomaterial-based combination therapies either combining a chemotherapy or DC immunotherapy with oAd ([34, 46], respectively) showed superior therapeutic effect than respective combination therapy in the absence of nanomaterial carrier, we foresee that combination of ICI and oAd could be of a great promise for effective cancer treatment.

### Enhancing viral replication and gene transfer of oAd through mild hyperthermia

Mild hyperthermia has been shown to enhance the sensitivity of cancer toward other treatment methods, such as chemotherapy, immunotherapy, and radiotherapy, and functions as a promising therapeutic adjuvant [49, 50]. The mild heat shock of oAd for the induction of hyperthermia using a pre-heated incubator led to greater viral burst and increased viral replication [51]. Based on these premises, Jung et al., proposed that laser irradiation-induced hyperthermia using a gold nanorod (GNR) could be a promising strategy to more precisely control heat shock to enhance the therapeutic potential oAd [52]. In this report, a combination of oAd expressing VEGF promoter-targeted artificial transcriptional repressor zinc-finger protein (RdB-K35/KOX) and PEGylated GNR was exposed to laser irradiation to induce hyperthermia (42 °C). Hyperthermia potentiated the cancer cell killing effect of 6 different oAds in vitro. The enhanced oncolytic effect by hyperthermia was due to improvement in the replication and endocytic uptake of virions into target cells. In specific, hyperthermia elevated the expression level of known regulators of endocytosis, such as clathrin, dynamin II, and caveolin I, resulting in improved cellular uptake, transgene expression, and viral replication of oAd. Importantly, the combination of RdB-K35/KOX, GNR, and irradiation (oAd + GNR + Laser) led to more potent tumor growth inhibition than naked RdB-K35/KOX or GNR plus laser irradiation during the entire observation period (up to 43 days post initial treatment); up to 2-fold more potent antitumor activity than monotherapy groups. The potent antitumor activity of oAd + GNR + Laser combination treatment was achieved by highest level of viral replication, VEGF suppression, and anti-angiogenic effect in tumor tissues. Their findings provide strong support that controlled hyperthermia by heat-generating nanomaterial may be a promising strategy to improve the therapeutic efficacy of oncolytic viruses in the future.

### Improving the therapeutic effect of other oncolytic viruses with polymer-based delivery system

Among several types of oncolytic viruses in clinical development, a vaccine strain of measles virus (MV) has been expected to induce strong antitumor effect in several clinical trials. Additionally, MV is internalized into cells through CD46 receptors, which are highly expressed in a wide range of cancer types, making it widely utilizable for cancer therapy. Although promising, the antiviral immune response and pre-existing immunity toward MV is of great concern toward the safety and potency of oncolytic MV. Nosaki et al., proposed that layer-by-layer deposition of ionic polymers onto the surface of oncolytic MV may address these inherent limitations of locally administered MV [53]. An oncolytic MV polyplex was formed by step-by-step addition of cationic PEI and anionic chondroitin sulfate (CS): a negatively charged MV is first reacted with cationic PEI to form a cationic MV/PEI complex, then the resulting product is bound to CS through electrostatic interactions and generates MV/PEI/CS particles. The MV/PEI/CS complex showed greater oncolytic activity than naked MV and greater resistance toward virus inactivation by anti-MV antibodies in vitro. Similar to the cases where oAd complexed with cationic nanomaterials overcomes CAR-dependent internalization in **Section 1** of the review, the oncolytic activity of MV/PEI/CS complex was inhibited to a lesser extent than naked MV in the presence of anti-CD46 antibodies, suggesting that the nanocomplex is likely internalized into cells independently of CD46. CD46-independent and unknown endocytosis mechanism of MV/PEI/CS complex can be concerning as it may lower cancer selectivity of the complex, thus further documentation and exploration of complex internalization is necessary in the future. Although reasons for enhanced internalization and potent oncolytic effect of the complex remains elusive, the authors suggest that potency of MV/PEI/CS may arise due to greater quantity of vrions being attached to plasma membrane of cancer cells than naked MV. Interestingly, this difference in anticancer effect between naked MV and MV/PEI/NS did not translate into in vivo, as both naked MV and MV/PEI/CS elicited similar antitumor effect in three different xenograft tumor models. However, the authors were able to show that MV/PEI/NS elicits more potent tumor growth inhibition than naked MV in a syngeneic murine tumor model where mice had been pre-immunized with MV prior to tumor inoculation, showing that polymer coating of oncolytic MV enables MV to retain their therapeutic efficacy against pre-immunized therapeutic targets.

One of the limitations as discussed in **Introduction** section for oncolytic virotherapy is limited penetration and distribution of the virus into tumor tissues. SELP hydrogel matrix was utilized to deliver oncolytic vaccinia virus following surgical resection of the tumors to eradicate any residual tumor cells and prevent tumor regrowth [54]. SELP hydrogel allowed more effective retention of infectious oncolytic vaccinia virus particles (up to 1000-fold higher retention than naked virion incubated in PBS for 7 days). The oncolytic vaccinia virus released from the gel was detectable up to 4 weeks of incubation, whereas the naked virions were not detectable after 1 week of incubation, suggesting that the protective gel matrix can preserve biological activity of oncolytic vaccinia virus in a similar manner as other gel depot systems utilizing oAds as described in **Section 3**. Furthermore, SELP-based delivery of oncolytic vaccinia virus led to higher level of transgene expression in tumor tissues than naked virus from 10 days to 25 days post treatment, showing that sustained release of oncolytic virus from protective gel can prolong the transgene expression of virion. Importantly, topical application of oncolytic vaccinia virus in SELP gel to high-volume residual tumors following incomplete surgical resection mimicking clinical scenarios (120 mm^3^ tumor volume) led to more than 2-fold greater transgene expression and more potent tumor growth inhibition than those of naked virus at day 12 and 25 post treatment, respectively. Interestingly, the authors have also explored the case of lower residual tumor volume (50 mm^3^) after surgical resection where they compared the therapeutic efficacy of oncolytic vaccinia virus in microscopic SELP gel particles with those retained in complete SELP gel, hypothesizing that microscopic gel particles with smaller particle diameters may enhance viral release from the gel and increase contact area with tumors. In line with their hypothesis, oncolytic vaccinia virus encapsulated in microscopic gel particles led to 50% higher transgene expression and more potent tumor growth inhibition than the virus applied with standard SELP gel. The mice treated with oncolytic vaccinia virus encapsulated in SELP particles also led to highest percentage of mice with complete tumor regression, suggesting that different preparation methods for virus encapsulation into gel matrix is of great importance to enhance the therapeutic potency of oncolytic virus against residual tumors that remain post-surgical resection.

## Conclusion

Oncolytic virotherapy is a highly promising therapeutic modality that is on the rise for cancer therapy with various products in clinical trials. Despite their promises, the therapeutic efficacy is not yet optimal and various strategies are actively being investigated to improve their therapeutic efficacy. To this end, a nanomaterial-based delivery platform can be a highly promising strategy to circumvent or overcome many of the limitations, such as rapid viral clearance, poor intratumoral retention, adverse inflammatory response, limited viral dispersion in tumor tissues, and nonspecific shedding of virion to the bloodstream or nontarget organs. Notably, these nanomaterials can be used as a platform to co-deliver other therapeutic modalities, such as chemotherapeutic and immunotherapeutics, in conjunction with oncolytic viruses to enhance the synergistic anticancer effect of multiple therapeutic modalities. As the field of nanomaterial-based delivery of oncolytic viruses is still in infancy, there are countless opportunities for product improvement and development to maximize the potential therapeutic benefits of oncolytic virotherapy.

## Data Availability

Not applicable.

## Abbreviations

ABP: Arginine-grafted bioreducible polymer
Ad: Adenovirus
Ad/DA3: DA3-coated Ad
Ad/PCION: Ad complexed with PCION
Ad/PPSA: Ad complexed with PPSA
APP: PEGylated PTX conjugated ABP
Arg: Arginine
CAR: Coxsackie and adenovirus receptor
CS: Chondroitin sulfate
DA3: Bile acid-conjugated PEI
DC: Dendritic cells
DWP418: Relaxing-expressing oAd
DWP418/gel: DWP418 loaded in gel
DWP418/PPSA: DWP418 complexed with PPSA
GHPA: Gelatin-hydroproxyphenyl propionic acid
GMCSF: Granulocyte macrophage colony stimulating factor
GNR: Gold nanorod
HmT: Hypoxia-responsive oAd
HmT-PCION: HmT complexed with PCION
ICI: Immune checkpoint inhibitors
IFN: Interferon
IL: Interleukin
MF: Magnetic field
MNP: Magnetic nanoparticle
MV: Measles virus
MV/PEI: MV complexed with PEI
MV/PEI/CS: MV/PEI complexed with CS
oAd + DC: Combination of oAd and DC
oAd + DC/gel: oAd and DC delivered by GHPA gel
oAd + GNR + Laser: Combination of RdB-K35/KOX with gold nanorod and laser
oAd: Oncolytic adenovirus
oAd-PB-Mag1: oAd complexed with PB-Mag1
oAd-PEI-Mag2: oAd complexed with PEI-Mag2
oAd-SO-Mag2: oAd complexed with SO-Mag2
oAd-TRAIL: TRAIL-expressing oAd
oAd-TRAIL/gel: oAd-TRAIL encapsulated in gelatin-based hydrogel
oAd-vp53 + PTX: combination of oAd-vp53 and PTX
oAd-vp53: oAd expressing a proapoptotic gene
oAd-vp53/APP: oAd-vp53 complexed with APP
PB-Mag1: polybrene-modified magnetic nanoparticles
PCION: PEGylated and cross-linked iron oxide nanoparticles
PEI: Poly(ethyleneimine)
PEI-Mag2: Lithium 3-[2-(perfluoroalkyl)ethylthio]propionate) complexed with 25 kDa branched PEI
PPSA: mPEG-PEI-g-Arg-S-S-Arg-g-PEI-mPEG
PTX: Paclitaxel
RdB-KOX: oAd expressing anti-angiogenic gene
RdB-KOX/DA3: RdB-KOX complexed with DA3
rPEI: Multidegradable bioreducible core-cross-linked PEI
SELP: Silk-elastin like protein polymer
shRNA: Short hairpin RNA
SO-Mag2: Silica-modified magnetic nanoparticles decorated with 25 kDa branched PEI
TRAIL: Tumor necrosis factor-related apoptosis-inducing ligand
VEGF: Vascular endothelial growth factor
